# Exploring the association between school-based peer networks and smoking according to socioeconomic status and tobacco control context: a systematic review

**DOI:** 10.1186/s12889-021-12333-z

**Published:** 2022-01-20

**Authors:** H. J. Littlecott, G. F. Moore, M. McCann, G. J. Melendez-Torres, L. Mercken, H. Reed, M. Mann, F. Dobbie, J. Hawkins

**Affiliations:** 1 Centre for Development, Evaluation, Complexity and Implementation in Public Health Improvement (DECIPHer), School of Social Sciences, Cardiff University, 1-3 Museum Place, CF10 3BD Cardiff, Wales, UK; 2 MRC/CSO Social and Public Health Sciences Unit, University of Glasgow, Berkeley Square, 99 Berkeley Street, Glasgow, G3 7HR UK; 3 Peninsula Technology Assessment Group (PenTAG), South Cloisters, University of Exeter, St Luke’s Campus, Heavitree Road, Exeter, EX1 2LU UK; 4 Department of Health Promotion, CAPHRI School for Public Health and Primary Care, Maastricht University, Maastricht, The Netherlands; 5 Specialist Unit for Review Evidence, Cardiff University, 6th Floor, Neuadd Meirionnydd, Heath Park Campus, CF14 4YS Cardiff, Wales, UK; 6 Usher Institute- University of Edinburgh, Doorway 1, Old Medical School, Teviot Place, Edinburgh, EH8 9AG UK

**Keywords:** Systematic review, Socioeconomic status, Inequality, Smoking, Smoking legislation, Social network analysis, Narrative review

## Abstract

**Background:**

Whilst prevalence of youth smoking in middle and high income countries has decreased, inequality has prevailed. The introduction of legislation regulating tobacco use in public spaces varies across countries, impacting the tobacco control context. Thus reviewing our knowledge of how social networks may influence smoking differently within different contexts is required to facilitate the development of context-specific interventions.

**Methods:**

The search, conducted on 31st May 2019, included the following smoking-related terms; schools, adolescents, peers and social networks. Inclusion and exclusion criteria were applied throughout the title and abstract screening and full text screening. Quality assessment and synthesis followed. Studies were narratively synthesised to identify changes according to legislative context. This synthesis was conducted separately for findings relating to three categories: socioeconomic status; social selection and influence; and network position.

**Results:**

Thirty studies were included. Differences in the relationship between network characteristics and smoking according to socioeconomic status were measured in five out of fifteen studies in Europe. Results varied across studies, with differences in network characteristics and their association with smoking varying both between schools of a differing and those of a similar socioeconomic composition. For studies conducted both before and after the introduction of comprehensive smoking legislation, the evidence for selection processes was more consistent than influence, which varied according to reciprocity. Findings showed that isolates were more likely to smoke and in-degree and out-degree centrality were related to smoking both before and after the introduction of legislation. The relationship between popularity and smoking was contingent on school level smoking prevalence in studies conducted before the introduction of legislation, but not after.

**Conclusions:**

Overall, effects according to socioeconomic status were underreported in the included studies and no consistent evidence of change after the introduction of a comprehensive smoking ban was observed. Further network analyses are required using more recent data to obtain a comprehensive understanding of how network processes may influence smoking differently according to socioeconomic status, and how adaptation could be used to enhance intervention effectiveness.

**Systematic review registration:**

International Prospective Register of Systematic Reviews (PROSPERO) registration number: CRD42019137358.

**Supplementary Information:**

The online version contains supplementary material available at 10.1186/s12889-021-12333-z.

## Background

In most high-income countries, smoking prevalence is at an all time low among young people. The Health Behaviour in School Aged Children study found that an average of 3% of 11 year olds and 11% of 13 year olds in European countries and Canada reported ever smoking in 2018, which reduced from 5 and 15% in 2014, respectively [1]. Despite this, smoking uptake remains a major public health concern internationally [2], with much adult smoking beginning in adolescence [3]. Moreover, whilst prevalence of smoking in middle and high income countries has decreased, inequality has prevailed [4], with prevalence decreasing less rapidly among disadvantaged groups [5]. In addition to this, whilst encouraged by the World Health Organization’s Framework Convention on Tobacco Control [6], legislation regulating tobacco use in public spaces varies across countries in terms of whether it has been introduced, when it was introduced and the level of coverage. This has an impact on the legal tobacco control context, as well as the political and sociocultural contexts [7] within each country and, therefore, the prevalence of and inequalities in smoking.

Social networks can be defined as connections between individuals or groups and the social structure that this creates can be measured empirically [8]. The link between peer network effects and adolescent tobacco smoking [9] and the influence of complex systems, such as schools, on tobacco smoking [10, 11] have been succesfully investigated within previous research employing social network analysis. Network effects can occur through social selection, social influence, homophily and network position. Social influence refers to the level to which an individual’s smoking behaviour is directly or indirectly influenced by their peers’ behaviour and/or attitudes, whereas social selection refers to an individual choosing friends according to whether they smoke or not. In this case smoking may initially drive friendship formation, before being reinforced through these friendships [12]. Homophily is defined as the extent to which individuals are similar to each other [13] and network position describes an individual’s position within a network, such as their level of popularity (centrality) [14], isolation or group (clique) membership [15]. A clique is an exclusive group of people who share interests, views, purposes, or patterns of behaviour. A liaison is a person who bridges communications between two or more groups. Isolates are those who do not actively participate in cliques or friendship groups [15]. Peer group structure refers to the regularised patterns of interactions among adolescents in a social system, such as density (the total number, compared to the total possible number of relationships in a network) [8]. These interactions characterise three major peer-defined social positions available to adolescents: clique member, liaison, and isolate. These are particularly pertinent for adolescent smoking, due to the increased importance of peer compared to parental approval among this age group [16]. A glossary of social network terms is provided in Additional file 1.

A systematic review by Seo & Huang [12] found that isolates were significantly more likely to smoke than clique members and that social selection was found to contribute more than social influence to subsequent adolescent smoking. However, to date reviews have not taken into account contextual issues, such as the legislative context in which the data were collected, and whether network effects may differ according to socioeconomic context.

Simulation models have estimated that intervention effects on smoking uptake can differ between schools, with effects moderated by school level smoking prevalence [17]. This suggests that it is plausible for the mechanisms by which social networks may influence smoking uptake in schools may differ according to school-level socioeconomic status (SES). This is contrary to previous research which has assumed these mechanisms, and the tendency for ‘popular’ students to be smokers, to be consistent across different settings [18]. This is particularly pertinent in light of the increasingly comprehensive tobacco control action in some countries over the past decade, which has accelerated overall denormalisation of smoking at the macro-systemic level, whilst inequality has prevailed [19].

However, a recent simulation study found that structural characteristics of a network, such as density and degree centrality, influenced the diffusion of network interventions, as well as their level of effectiveness [20]. Despite this, whilst intervention evaluations and designs are conducted within varied school and socioeconomic contexts, they do not tend to address these differential processes and outcomes [21].

In summary, reviewing our knowledge of how social networks may influence smoking differently within different contexts is required to facilitate the development of context-specific interventions.

Within this review the main focus will be upon the socioeconomic and legal context [7]. This review addresses the following research questions:

- What are the associations between school-based social networks and smoking/attitudes towards smoking among adolescents?
  - To what extent and how do these associations vary by SES, between countries, and over time?
  - To what extent and how do these associations vary according to the proximity of the introduction of comprehensive smoking legislation at the time of data collection?

## Methods

This review is reported in accordance with the reporting guidance the Preferred Reporting Items for Systematic reviews and Meta-Analyses (PRISMA) statement [22]. The review is registered with the International Prospective Register of Systematic Reviews (PROSPERO) (registration number: CRD42019137358) and the review protocol has been published in Systematic Reviews [23]. Quantitative and qualitative searches were conducted in parallel. This paper reports the results of quantitative review, whilst a further publication will follow to combine the quantitative and qualitative results. The information sources, search strategy and eligibility criteria were based on and extend those of Seo and Huang in their previous 2012 review [12].

### Information sources and search strategy

The search strategy included terms relating to smoking, schools, adolescents, peers and social networks and searched a variety of relevant databases and secondary sources. A glossary of social network terms is included in Additional file 1 and the Medline search strategy is included in Additional file 2. Further details on the databases searched are available in the review protocol [23].

### Eligibility criteria

Studies were included in the review if they met the following criteria. Papers which collected data from 1997 onwards, comprised secondary school students (age 11–18 years), school staff, parents or other education professionals, and focused on the whole population, or students of a low SES. Studies focused on special populations, such as those with special educational needs, were excluded. No language or geographical limits were set, but comparisons were made within the analyses according to whether the data were collected before or after the introduction of comprehensive smoking legislation, in each respective country, covering bans on smoking in all work places and public places, including restaurants and bars.

The search criteria have been guided by the Population Exposure Comparator Outcome (PECO) framework [24]. Further details are provided in the review protocol [23].

#### Screening, selection and data extraction

Two researchers (HL & HR) independently screened the titles and abstracts, followed by the full texts of each identified study using the inclusion and exclusion criteria. Discrepancies were resolved by a third researcher (GJMT). Data were then extracted, with authors contacted directly to request any information, such as the date of data collection, that was not reported. Extracted data for each study is included in Additional file 4 and further details of this process are available within the review protocol [23].

### Risk of bias (quality) assessment

Risk of bias assessment was undertaken independently for all included studies by two researchers (HL and HR). Discrepancies were resolved by a third reviewer before finalising quality assessments for papers (GJMT). Further details of the risk of bias assessment are reported within the review protocol [23].

### Synthesis

Studies were grouped according to both a priori defined groupings and those that emerged inductively as the data were analysed. These included the focus of network studies and whether data collection was conducted before or after the introduction of comprehensive tobacco control legislation, as a marker of the level of smoking normalisation. This synthesis was conducted separately for findings relating to three categories: socioeconomic status; social selection and influence; and network position. Due to the nature of social network data, whereby the parameters in network models are often specific to each study, a meta-analysis was not undertaken.

## Results

### Study selection

**Fig. 1:**
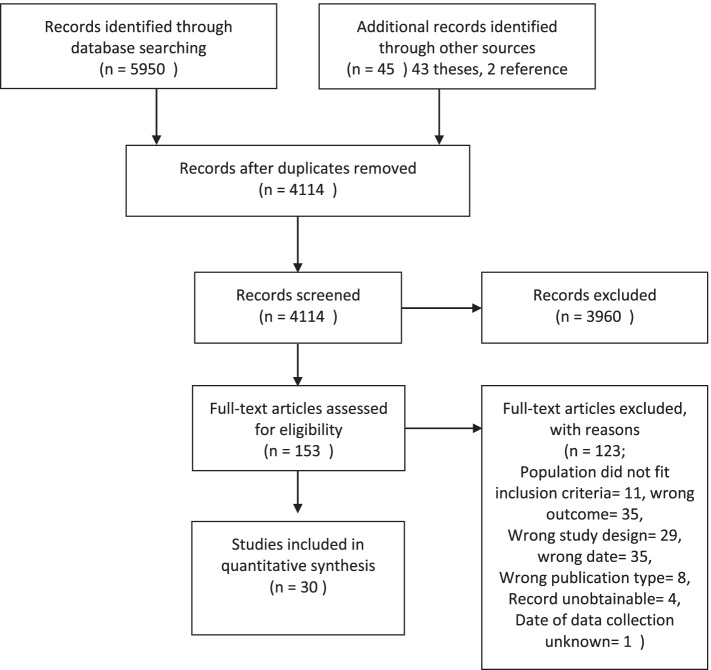
PRISMA flow diagram

The search identified 5950 records from databases, while 45 additional records were identified from other sources. After a comprehensive screening process, detailed in the PRISMA flowchart (Fig. 1), 30 studies were included in the systematic review.

### Study characteristics

All study characteristics are displayed in Tables 1 and 2.

**Table 1:** Characteristics of included studies

Larger study	Author and year	Year of data collection	Participant characteristics			Country	Study design	Software used	Analysis	Aim	Quality assessment	Synthesis category
			Age	Number of participants	Number of schools							
European Smoking Prevention Framework Analysis (ESFA)	Mercken et al. (2007) [25]	1998	12–13	1886	9	Netherlands	Longitudinal	Mplus 4.1	Structural Equation Modelling (SEM)	To examine the effect of influence and selection for reciprocal and non-reciprocal friendship on smoking	Medium	Social selection and influence
	Mercken et al. (2009a) [26]	1998	12–13	1886	9	Netherlands	Longitudinal	Mplus 4.1	SEM	To examine the specific contribution of influence and selection for reciprocal and non-reciprocal friendship and deselection on smoking changes.	Medium	Social selection and influence
	Mercken et al. (2009b) [27]	1998	Mean 13	7704	17 Danish, 11 Finnish, 9 Dutch, 8 Portugese, 4 UK & 21 Spanish	Denmark, Finland, Netherlands, Portugal, UK, Spain	Longitudinal	SIENA	Stochastic Actor Oriented Model (SAOM)	To examine smoking-related friendship selection and friends’ influence within the same school grade, while controlling for alternative selection mechanisms.	Medium	Social selection and influence
	Mercken et al. (2010a) [28]	1998	13–16	1326	11	Finland	Longitudinal	SIENA	SAOM	To examine the strength of influence and selection processes on smoking for reciprocal and non-reciprocal friendship	High	Social selection and influence
	Mercken et al. (2010b) [29]	1998	13–16	1163	9	Finland	Longitudinal	SIENA	SAOM	To examine gender differences in the strength of influence and selection processes on smoking for reciprocal and non-reciprocal friendship	High	Social selection and influence; Network position
Teenage Health in Schools (THiS) study	Turner et al. (2006) [30]	2001	13–15	489 baseline, 407 follow-up	2	Scotland	Cross-sectional	NEGOPY 4.50, SPSS	×2 test and F ratio (not multivariate)	To investigate whether peer structures and influences affect smoking rates	Low	Socioeconomic status; Network position
	Pearson et al. (2006) [31]	2001	13–15	3379	9	Scotland	Cross-sectional	NEGOPY	Logistic regression	Do associations between network measures and substance use differ according to context	Low	Socioeconomic status; Network position
ASSIST- A Stop Smoking In Schools STudy	Steglich et al. (2012) [32]	2001	12–16	596 baseline, 585 follow-up	3	UK	Longitudinal	SIENA	SAOM	To compare results of different approaches to SABM in measuring link between network structure and smoking	Medium	Social selection and influence
	Mercken et al. (2012) [33]	2001	12–14	1677 baseline, 1614 follow-up	11	UK	Longitudinal	SIENA	SAOM	To examine how smoking based selection and influence processes change over time	High	Social selection and influence
Promoting School-Community-University Partnerships to Enhance Resilience (PROSPER)	Copeland et al. (2017) [34]	2002	13–18/19	11,802	28 school districts	USA (Iowa)	Longitudinal	Not specified	Autoregressive Latent Trajectory Models (ALT)	To examine whole and ego network effects on smoking, particularly isolation	Medium	Network position
	Ragan (2016) [35]	2002	13–18/19	Mean 6200 at each wave	27 school districts	USA (Iowa)	Longitudinal	SIENA	SAOM	To examine the effect of peer beliefs on smoking-	Medium	Social selection and influence
	McMillan et al. (2018) [36]	2002	13–18/19	9135	51	USA (Iowa)	Longitudinal	SIENA	SAOM	To investigate the effect of gender on peer influence and selection	High	Social selection and influence
	Osgood et al. (2014) [37]	2002	11–14	9500 at each wave	27 (rural, low SES)	USA (Iowa)	Longitudinal	HLM 6.08	Multi-level regression	To examine network positive in cohesive peer groups and its association with substance use	Medium	Network position
Context of Adolescent Substance Abuse study	Ennet et al. (2008 [38])	2002	11–17	6579	13 middle schools W1, 18 high schools W2/3	USA (North Carolina)	Longitudinal	SAS V9	Hierarchical Growth Models (HLM)	To investigate peer networks and context for substance abuse	Medium	Social selection and influence; Network position
	Ennet et al. (2006) [39]	2002	11–17	5104	13 middle schools W1, 18 high schools W2/3	USA (North Carolina)	Longitudinal	SAS IML, UCINET, HLM	Hierarchical Generalized Linear Models (HGLM)	To investigate peer networks and context for substance abuse	Medium	Network position
FINEdu (Finnish Educational Transitions)	DeLay et al. (2013) [40]	2004	15–17	1419	9 (4 vocational, 5 academic)	Finland	Longitudinal	SIENA	SAOM	To investigate the effect of selection, deselection and socialisation on smoking	High	Social selection and influence
	Kiuru et al. (2010) [41]	2005	15–18	1419	9	Finland	Longitudinal	RSIENA	SAOM	To examine changes in smoking in relation to changing or stable peer groups	High	Social selection and influence
Unnamed study	Huisman & Bruggeman (2012) [42]	2008	13–14	961	5	Netherlands	Longitudinal	RSIENA	SAOM	To examine how networks mediate the relationship between smoking and SES	Medium	Socioeconomic status; Social selection and influence
	Huisman (2014) [43]	2008	13–14	857	4	Netherlands	Longitudinal	RSIENA	SAOM	To examine the link between network and smoking while accounting for selection effects	Medium	Social selection and influence
SILNE (Smoking Inequalities – Learning from Natural Experiments)	Lorant et al. (2017) [44]	2013	14–16	10,604	50	Europe (6 countries)	Cross-sectional	SAS 9.3	Logistic regression	To investigate the role of social ties in socioeconomic differences in smoking	Medium	Socioeconomic status
	Robert et al. (2019) [45]	2013	14–17	11,015	50	Europe (6 countries)	Cross-sectional	SAS 9.3	Multi-level logistic regression	To investigate the association between academic performance, smoking and SES	Medium	Socioeconomic status
	Mulassi et al. (2012) [46] (cross-sectional)	2010	14–18	285	1	Argentina	Cross-sectional	Pajek, Epi info, SPSS	Kamada-Kawai algorithm	To study the association between network structure and smoking	Low	Network position
	Valente et al. (2013) [47]	2010	15–16	1707	5	USA (LA)	Cross-sectional	Not specified	Exponential Random Graph Models (ERGMS)	To compare the association between adolescent smoking and friend smoking across different types of network	Medium	Social selection and influence
	Forster et al. (2015) [48]	2012	12–14	184	1	USA (LA)	Cross-sectional	UCINET, Stata	Logistic regression	To investigate the interplay of individual characteristics and peer influences on substance use	Low	Network position
	Hall & Valente (2007) [49]	2001	11–13	1960 baseline, 880 follow-up	6	USA (LA)	Longitudinal	Stata and LISREL	SEM	To evaluate the relative strength of selection and influence on adolescent smoking over two timepoints	Medium	Social selection and influence
	Ramirez-Ortiz et al. (2012) [50]	2003	15–19	486 baseline, 399 follow-up	1	Mexico	Longitudinal	NetMiner II 2.4.0, SPSS, Stata	Chi squared and logistic regression	To investigate the effect of centrality on smoking	Low	Network position
	Lakon & Valente (2012) [51]	2004	12–21 (97% 12–18 years old)	851	14	USA (LA)	Cross-sectional	SAS	HLM	To investigate social integration and smoking	Medium	Social selection and influence
	Van Ryzin et al. (2016) [52]	2000	11–14	1289	8	USA (Pacific Northwest)	Longitudinal	RSIENA	SAOM	To investigate whether being well-liked can serve as a risk factor for substance use	Medium	Network position
	Valente et al. (2005) [18]	2001	10–12	1486	16	USA (LA)	Longitudinal	Not specified	Multi-level logistic regression	To investigate popularity, network position and smoking	Medium	Network position
	Kobus & Henry (2010) [53]	1997	11–14	163	1	USA (Illinois)	Cross-sectional	FNET	Generalised Linear Models	To investigate the effect of network position, peer substance use and their interaction on adolescents’ own use	Medium	Network position

**Table 2:** Details of measures and smoking legislative context for included studies

Larger study	Author and year	Socioeconomic status measure	Social network measure	Network boundary	Smoking measure	Conducted before/after introduction of comprehensive smoking ban	Country and year of smoking ban
European Smoking Prevention Framework Analysis (ESFA)	Mercken et al. (2007) [25]	N/A	Nominate up to five friends	Friends inside and/or outside school	Weekly smoking behavior	Before (−9 years)	Netherlands 2008
	Mercken et al. (2009a) [26]	N/A	Nominate up to five friends	Friends inside and/or outside school	Weekly smoking behavior	Before (−9 years)	Netherlands 2008
	Mercken et al. (2009b) [27]	N/A	Nominate up to five friends	Friends inside and/or outside school	Weekly smoking behavior	Before (−8 years or more)	Denmark, no comprehensive ban, Finland 2006, Netherlands 2008, Portugal 2007, UK (England) 2007
	Mercken et al. (2010a) [28]	N/A	Nominate up to five friends	Friends inside and/or outside school	Weekly smoking behavior	Before (−7 years)	Finland 2006
	Mercken et al. (2010b) [29]	N/A	Nominate up to five friends	Friends inside and/or outside school	Weekly smoking behavior	Before (−7 years)	Finland 2006
Teenage Health in Schools (THiS) study	Turner et al. (2006) [30]	N/A	Nominate up to six friends	Unclear, but analysis focuses on friends inside the school	Ever smoking and smoking frequency	Before (−5 years)	Scotland, UK 2006
	Pearson et al. (2006) [31]	School level: Proportion of pupils in receipt of a clothing grant.	Nominate up to six friends	Unclear, but analysis focuses on friends inside the school	Ever smoking and smoking frequency	Before (−5 years)	Scotland, UK 2006
ASSIST- A Stop Smoking In Schools STudy	Steglich et al. (2012) [32]	Individual level: Family Affluence Scale; School level: free school meal entitlement	Nominate up to six friends	School year group	Ever smoking and smoking frequency	Before (−5 to 6 years)	England and Wales, UK 2006–2007
	Mercken et al. (2012) [33]	Individual level: Family Affluence Scale; School level: free school meal entitlement	Nominate up to six friends	School year group	Ever smoking and smoking frequency	Before (−5 to 6 years)	England and Wales, UK 2006–2007
Promoting School-Community-University Partnerships to Enhance Resilience (PROSPER)	Copeland et al. (2017) [34]	School level: free school meal entitlement	Nominate up to seven friends. Report how many close friends they have in other year groups and schools	School year group	Ever smoking and smoking frequency	Before (−6 years)	USA (Iowa) 2008
	Ragan (2016) [35]	N/A	Nominate up to seven friends	School year group	Ever smoking and smoking frequency and beliefs about smoking	Before (−6 years)	USA (Iowa) 2008
	McMillan et al. (2018) [36]	N/A	Nominate up to seven friends	School year group	Ever smoking and smoking frequency	Before (−6 years)	USA (Iowa) 2008
	Osgood et al. (2014) [37]	N/A	Nominate up to two best friends and five additional friends	School year group	Smoking frequency	Before (−6 years)	USA (Iowa) 2008
Context of Adolescent Substance Abuse study	Ennet et al. (2008) [38]	Individual level: parental education	Nominate up to five friends	School year group	Smoking frequency	Before	USA (North Carolina), No comprehensive ban
	Ennet et al. (2006) [39]	N/A	Nominate up to five friends	School year group	Smoking frequency	Before	USA (North Carolina), No comprehensive ban
FINEdu (Finnish Educational Transitions)	DeLay et al. (2013) [40]	Individual level: parental education	Nominate up to three friends	School year group	Ever smoking and smoking frequency	Before (−2 years)	Finland 2006
	Kiuru et al. (2010) [41]	N/A	Nominate up to three friends	School year group	Smoking frequency	Before (−1 years)	Finland 2006
Unnamed study	Huisman & Bruggeman (2012) [42]	Individual level: parental education; School level: school type	Nominate up to 15 friends	School year group	Smoking frequency and quantity	Same year	The Netherlands 2008
	Huisman (2014) [43]	N/A	Nominate up to 15 friends	School year group	Smoking frequency and quantity	Same year	The Netherlands 2008
SILNE (Smoking Inequalities – Learning from Natural Experiments)	Lorant et al. (2017) [44]	Individual level: parental education, family affluence, subjective social status, parental working status and housing ownership	Nominate up to 5 friends	Two school year groups	Ever smoking, smoking frequency and nicotine dependence	After (+ 2 to + 8 years)	Europe (Belgium 2011, Finland 2008, Germany 2007, Italy 2005, Netherlands 2008, Portugal 2007)
	Robert et al. (2019) [45]	Individual level: parental education	Nominate up to 5 friends	Two school year groups	Smoking frequency	After (+ 2 to + 8 years)	Europe (Belgium 2011, Finland 2008, Germany 2007, Italy 2005, Netherlands 2008, Portugal 2007)
	Mulassi et al. (2012) [46] (cross-sectional)	N/A	Nominate up to 10 friends	Whole school	Ever smoking and smoking frequency	Before (−3 years)	Argentina 2013
	Valente et al. (2013) [47]	Individual level: reduced or free lunch	Nominate up to 7 best friends	Completed 3 times, bounded by classroom, school year group and unbounded	Ever smoking, smoking frequency and intention	After (+ 12 years)	USA (LA) 1998
	Forster et al. (2015) [48]	Individual level: median household income	Nominate up to 5 best friends	Whole school	5 items measuring lifetime smoking	After (+ 14 years)	USA (LA) 1998
	Hall & Valente (2007) [49]	Individual level: ethnicity and number of rooms in house	Nominate up to 5 friends	Classroom	Ever smoking and smoking intention	After (+ 3 years)	USA (LA) 1998
	Ramirez-Ortiz et al. (2012) [50]	N/A	Nominate up to 6 friends	Whole school	Ever smoking and current smoking	Before	Mexico (no comprehensive ban)
	Lakon & Valente (2012) [51]	N/A	Nominate up to 5 best friends	Classroom	Past month smoking frequency	After (+ 6 years)	USA (LA) 1998
	Van Ryzin et al. (2016) [52]	N/A	Nominate unlimited friends who they would like to be in a group	School year group	Past month smoking frequency	Before (−5, −9 and no comprehensive ban)	USA (Pacific Northwest; Idaho no comprehensive ban, Oregon 2009, Washington 2005)
	Valente et al. (2005) [18]	N/A	Nominate up to 5 closest friends	Classroom	Ever smoking and smoking susceptibility	After (+ 3 years)	USA (LA) 1998
	Kobus & Henry (2010) [53]	N/A	Nominate up to 6 friends	3 school year groups	Smoking frequency	Before (−11 years)	USA (Illinois) 2008

#### Context

Studies were categorised according whether data were collected before or after the introduction of comprehensive legislation banning smoking in all public indoor spaces, including bars and restaurants in the context being studied. Twenty one of the studies included in this review collected data before such legislation was introduced, whilst nine studies were conducted after. Nine European countries, the United States of America, one Central American and one South American country were represented.

#### Study design

Nineteen studies employed a longitudinal design, whilst nine employed a cross-sectional design. The number of schools in the included studies ranged from one to 51.

#### Social network methods

Studies used a variety of social network methods. Twelve employed Stochastic Actor-Oriented Model (SAOM). This method is interchangeably referred to as both Stochastic Actor-Based Models (SABM) and Stochastic Actor-Oriented Models (SAOM) in the literature. To avoid confusion, SAOM will be used consistently in the text to describe this method. SAOMs are longitudinal, actor-oriented modelling methods which were conceived in 1996 [54], but not used within the social network and adolescent smoking literature until 2009. This means that many studies have retrospectively analysed older datasets using this method. Other analyses employed regression modelling [4], multilevel modelling [3], structural equation modelling [3], exponential random graph modelling [1], chi-squared [1] and longitudinal modelling [5]. One study solely visualised networks using the Kamada-Kawai algorithm.

#### Risk of bias (quality) assessment

Overall, five studies were rated low, 19 studies were rated medium, and six studies were rated high quality. Details of the quality assessment are outlined in Additional file 3.

#### Focus of included studies

**Fig. 2:**
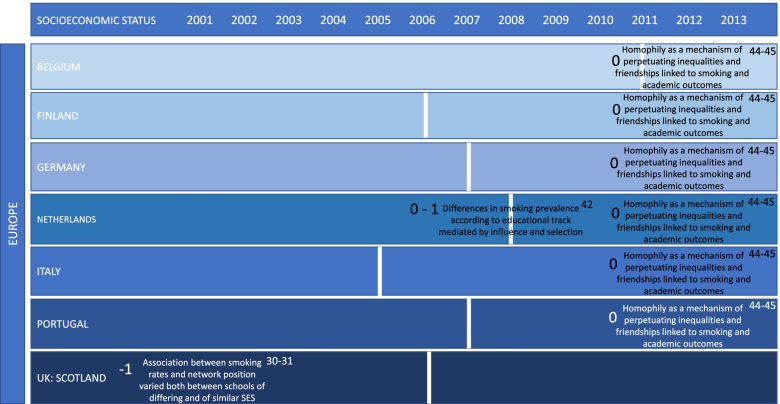
Summary of study findings relating to socioeconomic status according to year of publication and country. Vertical white lines represent the introduction of a comprehensive smoking ban in each country. Numbers, − 1, 0 or 1, to the left of each set of results refer to the study quality ratings of low, medium and high, respectively. Superscript numbers reference the study that each set of results refer to

**Fig. 3:**
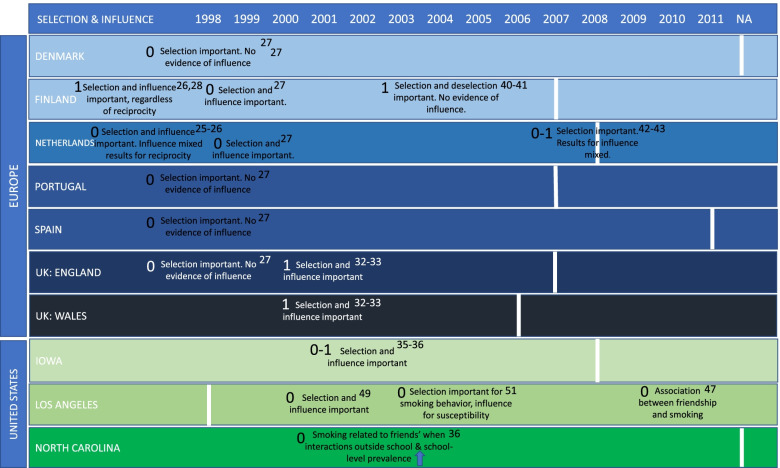
Summary of study findings relating to selection and influence according to year of publication and country. Vertical white lines represent the introduction of a comprehensive smoking ban in each country. -1, 0 or 1 refer to the quality ratings of low, medium and high, respectively. Superscript numbers reference the study that each set of results refer to

**Fig. 4:**
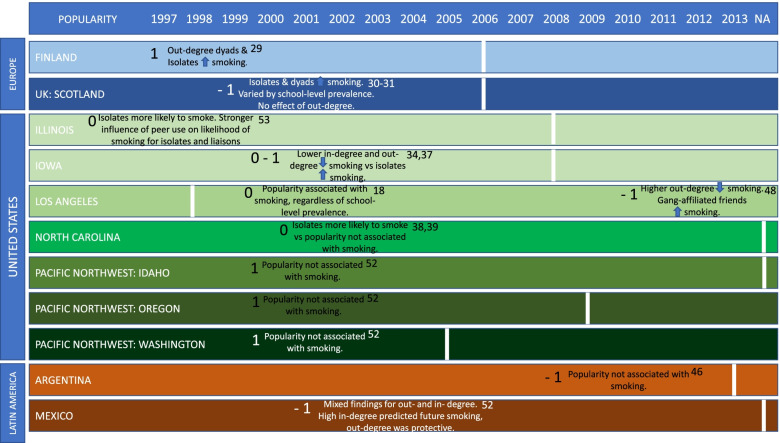
Summary of study findings relating to network position according to year of publication and country. Vertical white lines represent the introduction of a comprehensive smoking ban in each country. -1, 0 or 1 refer to the quality ratings of low, medium and high, respectively. Superscript numbers reference the study that each set of results refer to

The network characteristics measured and associated with adolescent smoking varied across studies. Pupil level characteristics included centrality (popularity), homophily (i.e. level of similarity between alters of characteristics, such as gender or socioeconomic status) and isolation. Social level characteristics included best friend smoking, peer beliefs, social selection, social influence, gang-affiliated friends, peer pressure and transitive triad membership. System level characteristics included school-level smoking prevalence, density and time with friends outside of school. The key findings are reported in Figs. 2, 3 and 4 for socioeconomic status; selection and influence; and network position. Results are placed along a timeline showing their placement by date and presence of a smoking ban.

### Findings focused on socioeconomic status

Differences in the relationship between network characteristics and smoking according to SES were measured in five out of fifteen studies in Europe. No studies outside of Europe considered differences according to SES. Out of the studies focused on SES, two collected data prior to the introduction of a comprehensive smoking ban [30, 31] and three after [42, 44, 45].

### Socioeconomic status

#### Studies conducted before the introduction of a comprehensive smoking ban

The two studies conducted before the introduction of a comprehensive smoking ban were rated as low quality and provided evidence that the association between smoking rates and network position varied between schools of differing SES composition [30]. Variance was also observed between schools of similar socioeconomic composition [31].

One study found that the link between network position and smoking varied between two schools of a low socioeconomic composition [30]. Within both schools, isolates and dyads were more likely to be smokers. However, one school observed no difference for popularity, whilst the other observed that no popular students were smokers [30]. Another study compared effects between eight schools of a low and high SES finding that popular students attending more affluent schools were more likely to smoke [31].

### Socioeconomic status

#### Studies conducted after the introduction of a comprehensive smoking ban

Studies conducted after the introduction of a comprehensive smoking ban were rated as medium quality and showed that individuals from a lower socioeconomic background were more likely to smoke [44, 45]. It was also demonstrated that homophily on the basis of SES may be a mechanism for perpetuating inequalities in smoking, through higher exposure to friends and families from a lower socioeconomic background, who are more likely to smoke [44]. In addition, one study found that friendships were related to smoking which may in turn be linked to academic outcomes, with those who smoke more likely to achieve lower academic outcomes [45].

A further study in the Netherlands in 2008, which was conducted in the same year as the introduction of comprehensive smoking legislation and rated as medium quality, focused on differences between students’ educational track [42]. Findings showed that differences in smoking prevalence according to educational track were largely mediated by the percentage of friends who smoke and friend influence and selection.

### Socioeconomic status: summary

Overall, students from a lower SES background were more likely to smoke and to be exposed to others’ smoking. Variance in network characteristics and their association with smoking varied both between schools of differing and those of similar socioeconomic composition. Differences in findings before and after the introduction of a comprehensive smoking ban were not evident within the available data.

### Overall findings

#### Social selection and influence

Sixteen studies focused on selection and influence, with 12 of these conducted before and four of these conducted after the introduction of a comprehensive smoking ban. Figure 3 shows the key findings for selection and influence on a timeline according to which country/region they originate from and when data were collected in relation to the introduction of comprehensive smoking legislation (represented by the white, vertical lines). Where the white vertical line is placed under NA (Not Applicable), this demonstrates that there is no current comprehensive smoking legislation in place. All studies measuring selection and influence were given the rating of either medium or high quality.

### Social selection and influence

#### Studies conducted before the introduction of a comprehensive smoking ban

Studies conducted before the introduction of a comprehensive smoking ban consistently found evidence for both selection and influence, although these varied by reciprocity and analysis method [25, 26, 28, 29, 32, 33, 35, 36, 38]. Only two studies from Finland [40, 41], both rated as high quality, and a cross-country comparison of six European countries [27], rated as medium quality, produced contrary results.

Five studies analysed data from the European Smoking Prevention Framework Analysis (ESFA). Four studies analysed data collected in the Netherlands [25, 26], rated medium quality, and Finland [28, 29], rated high quality, in 1998. Findings varied by analysis method. For example, studies that employed Stochastic Actor-Oriented Models the found that smoking similarity selection and influence were important for both reciprocal and non-reciprocal friendships [28, 29], whereas findings for influence varied according to reciprocity in studies employing structural equations modelling. A further study looked across six European countries; Denmark, Portugal, Spain, the Netherlands, Finland and the UK. Findings from this study demonstrated smoking similarity selection processes were stronger than influence processes. All six countries showed evidence of selection, but evidence of influence was only found in Finland and the Netherlands [27].

Four further studies employed Stochastic Actor-Oriented Models using data from two separate studies. The A Stop Smoking In Schools Trial (ASSIST) [32, 33], rated high and medium quality respectively, and the PROSPER Partnership Model [35, 36], rated high and medium quality. All studies found positive and significant relationships between smoking and both influence and smoking similarity selection.

A further study, rated medium quality, accounted for interactions outside of school using hierarchical growth models on data from the Context of Adolescent Substance Use Study in the US. They found that the likelihood of smoking relating to friends’ smoking increased with higher interactions outside of school and as school-level smoking prevalence increased [38].

In contrast to the results above, two studies analysed FINedu data from Finland using actor oriented models [40, 41]. Both found evidence of peer smoking similarity selection and deselection, whereby individuals decide to no longer be friends with those who do not match their smoking behaviour, but not influence. Selection effects were strongest within low smoking groups, whereas smoking-similarity deselection effects were strongest among high smoking groups.

### Social selection and influence

#### Studies conducted after the introduction of a comprehensive smoking ban

Studies conducted after the introduction of a comprehensive smoking ban were mixed. One study, rated medium quality, observed both effects of selection of smokers as friends and influence [49]. Although influence was more nuanced according to reciprocity, with those who had been identified as friends by smokers, but who did not reciprocate, being less likely to smoke. A further study by Lakon and Valente [51], rated medium quality, also found that the selection of smoker friends directly influenced later smoking behaviour, yet found more nuanced findings for influence. Findings showed that influence processes may indirectly affect smoking susceptibility through shaping the peer environment [51]. The other two studies by Huisman, rated medium quality, employed SAOMs using the same dataset and observed smoking similarity selection effects, but evidence of social influence was mixed [42, 43]. Huisman & Bruggeman [42] found evidence of social influence, whilst Huisman [43] found no evidence for the influence of friends’ smoking behaviour, but did observe positive influence effects for friends’ attitudes towards smoking [43].

A further study, rated medium quality, employed ERGMs to measure associations rather than selection or influence, finding evidence to support the association between friendship with smokers and an increased likelihood of individual smoking [47].

### Social selection and influence: summary

In summary, for studies conducted both before and after the introduction of comprehensive smoking legislation, the evidence for selection processes was more consistent than influence, which varied according to reciprocity.

### Network position

Fourteen studies focused on network position, with 12 of these conducted before and two of these conducted after the introduction of a comprehensive smoking ban. Figure 4 shows the key findings for network position on a timeline according to which country/region they originate from and when data were collected in relation to the introduction of comprehensive smoking legislation (represented by the white, vertical lines).

### Network position

#### Studies conducted before the introduction of a comprehensive smoking ban

Studies conducted before the introduction of a comprehensive smoking ban that measured popularity showed mixed findings. Five studies identified isolates [29, 31, 37, 39], two rated medium, one high and one low quality, whilst one identified liaisons [53], rated medium quality, as those students most likely to smoke. A further study found that the positive association between measured peer cigarette use and an individuals’ likelihood to smoke was stronger for isolates and members, whilst the positive association between perceived peer use and an individuals’ likelihood to smoke was stronger for members of cliques [53].

Five studies found in-degree centrality (popularity relating to the number of people who have nominated each individual as a friend) to be related to smoking [34, 41, 50], rated high, medium and low. One study broke this down by school type according to low and high smoking prevalence, and found that the school with a high smoking prevalence showed no difference, whereas in the school with a low smoking prevalence, popular students were less likely to be smokers [30]. This study was rated low quality. Three studies related out-degree centrality (popularity relating to the number of people nominated as a friend by each individual) to smoking [29, 34], with one showing it to have a protective effect [50]. Whilst two studies found no association with smoking and out-degree centrality [31, 41].

In contrast three studies did not find an association between popularity and smoking, instead finding evidence of a link between homophily [46, 52], rated low and medium quality, prevalence [38, 52] and betweenness centrality [38].

### Network position

#### Studies conducted after the introduction of a comprehensive smoking ban

For the two studies conducted after the introduction of a comprehensive smoking ban, one found an association between in-degree centrality (popularity) and smoking, whilst the other identified out-degree centrality to have a protective effect against smoking.

Valente [18] used multi-level logistic regression to investigate the link between in-degree, classroom-based popularity, network position and smoking, in California, US, this study was rated medium quality. They found that popular students were more likely to smoke and to be susceptible to smoking and that this was found within schools with both a low and high smoking prevalence. Betweenness centrality, closeness and integration were also associated with an increased likelihood of smoking. When measuring out-degree centrality, individuals who named more friends were less likely to smoke.

Forster et al. [48] used data from the US, finding that those with higher out-degree were less likely to smoke tobacco, whereas those with gang-affiliated friends were more likely to, this study was rated low quality.

### Network position: summary

In summary, isolates were more likely to smoke and both in-degree and out-degree centrality were related to smoking both before and after the introduction of comprehensive smoking legislation. Findings relating to popularity varied according to temporal context, with the relationship between popularity and smoking contingent on school level smoking prevalence in studies conducted before the introduction of comprehensive smoking legislation, but not after.

## Discussion

This paper presented a comprehensive review of the literature on social network mechanisms relating to adolescent smoking, and how the findings varied according to country, time and the introduction of comprehensive national tobacco policies.

### Socioeconomic status

Overall, findings showed that effects according to SES are underreported in studies investigating school-based social networks and their influence on smoking. The finding that popular students from more affluent schools were more likely to smoke [31] contrasts with what would be expected from previous findings, where schools with a higher smoking prevalence are more likely to have a lower socioeconomic composition [55, 56]. Alexander et al. [56] found that popularity significantly interacted with school level smoking prevalence in relation to adolescent smoking, with popular students less likely to smoke in schools with a low prevalence. Moreover, Fletcher & Bonell [55] hypothesised that the processes through which substance use diffuses through peer networks differ between schools of varying SES. Within a more affluent case study school, the authors describe a marginalisation of more deprived students from school culture, leading to formation of counter-school sub-cultures. Within a more deprived case study school however, mainstream school peer culture was framed more strongly around substance use. This study sets up a plausible, but as yet untested, hypothesis that could be applied to smoking. Findings from the study which analysed separately by academic and vocational tracks within the same schools [42] were more in line with this, whereby smoking was more acceptable and mainstream behaviour within the vocational, compared to academic, track. This suggests that the network effects occurring as a result of being segregated may perpetuate inequalities in smoking prevalence. Indeed, students on the vocational track have been shown to have a higher smoking prevalence and an awareness among students of lower future prospects [57]. In order to more fully explore this hypothesis, further research must make use of SNA in a greater number of schools, so that we can identify generalisable trends.

It is also highly important to investigate other network-related dimensions of difference alongside SES. For example, network effects may operate in a different manner according to whether they have relatively stable or transient populations [58]. This is in line with the findings from the current review which revealed differing results both between schools of varied and schools of a similar socioeconomic composition [30, 31]. Further, findings showed that students from a lower SES were more exposed to smoking and therefore more likely to smoke, in poorer communities where smoking remains normalised at the ‘meso-level’ [44, 45]. This is supported by previous intervention research. For example, in 2001 A Stop Smoking In Schools Trial (ASSIST) harnessed peer influence to prevent smoking. Findings showed a higher level of intervention effectiveness in schools with a higher number of students from a lower socioeconomic background, more stable populations within close-knit communities, higher smoking rates and higher social network density (actual number of ties in relation to potential ties) [58]. Further research is required using more recent data to understand how peer influence mechanisms differ between school contexts [7]. The finding within the current review that differences before and after the introduction of a comprehensive smoking ban were not evident, may have been due to the heterogeneity of study designs and definition and measurement of SES across these two categories as well as the higher level of quality attributed to the later studies.

### Social selection and influence

For studies conducted both before and after the introduction of comprehensive smoking legislation, the evidence for selection processes was more consistent than influence, which varied according to whether this was measured for reciprocal or non-reciprocal ties [25, 26, 28, 29, 32, 33, 35, 36, 38, 51]. This may suggest that smoking-related selection and deselection play a key role in friendship formation within the school setting and, thus, in harnessing peer influence to prevent or stop smoking. This aligns with previous research showing that peer groups have an impact on smoking behaviour with more consistent evidence of social selection over social influence [12, 59].

Findings showed consistent evidence for social influence before, but mixed findings after the introduction of comprehensive smoking legislation. Thus, in line with previous evidence from ASSIST [58], suggesting social influence on adolescent smoking may be weaker within a context where smoking is denormalised. Data from the Finedu study, collected in 2004 [40] and 2005 [41] before the introduction of a comprehensive smoking ban, found evidence of selection, but not influence. This may be due to these data being collected in Finland, where laws outlining strict smoking restrictions were put in place in 1995. This was not categorised as a comprehensive smoking ban for the purpose of this review as legislation did not include bars and restaurants until 2007. This may indicate stronger anti-smoking norms in this country at the time of data collection compared with the other studies conducted before the introduction of a comprehensive smoking ban [60].

Within this review no change over time was observed in studies measuring selection and influence, despite smoking becoming increasingly denormalised within most western countries throughout the time period of data collected by included studies, 1997 onwards [6]. This may be attributed to a lack of studies which focus on more recent data.

### Network position

Many studies within this review found that isolates were more likely to smoke. This is consistent with a previous systematic review [12], which found isolates were more likely to smoke and with previous research, which has consistently demonstrated an association between loneliness and smoking [61]. However, this sits in contrast to both the literature reviewed in this review and previous research findings outlined above, demonstrating more consistent evidence of social selection over social influence on smoking [59].

The current review showed the relationship between popularity and smoking to be contingent on school level smoking prevalence in studies conducted before the introduction of comprehensive smoking legislation, but not after. These later studies contrast with previous evidence on the determinants of adolescent smoking which show smoking to be a key determinant of popularity [51, 56] and which has assumed these mechanisms, and the tendency for ‘popular’ students to be smokers, to be consistent across different settings [18]. This demonstrates the importance of further interrogating and understanding social network processes over and above the determinants of smoking and analysing how these results may differ according to school context [62]. In addition, the finding in the current review that later studies did not observe differences according to popularity may be in part due to the denormalisation of smoking after the introduction of comprehensive smoking legislation [2], thus reducing the incentive to smoke in order to engage in an activity which is perceived as ‘cool’ and or socially normative.

### Strengths and limitations

Strengths of this review include the conduct of a comprehensive search of the published and grey literature, as well as the combination of studies with differing methods of SNA, samples and contexts. Whilst it was a challenge to synthesise and compare across studies, it allowed for the inclusion of a wider range of studies and provided a richer understanding of the existing literature.

Only two studies outside of Europe of the United States were eligible for inclusion within this review, suggesting that further research is required to understand social network processes within a wider variety of countries. Both studies that were conducted in Central or South America were conducted at a later date, but were still conducted prior to the introduction of a smoking ban, in a context where smoking was more normalised [46, 50].

Studies varied widely according to how they defined smoking status. Thus, these differences were not accounted for within the review as making direct comparisons between such studies was challenging.

The latest date of data collection of studies in this review was 2013, and most countries introduced comprehensive smoking bans between 2005 and 2008. Most studies analysed data collected before the introduction of a comprehensive smoking ban, making it difficult to compare across contexts and, whilst methodological advances have occurred, they have mainly been used to reanalyse older data sets rather than providing greater insight into more recent contexts. Rather, the absence of this data was an important finding in itself. This may indicate that a longer period of study is required before significant changes can be observed in the social network processes that are associated with smoking among adolescents. This is consistent with complex systems thinking whereby it takes years for new practices to embed within a system and emergent phenomena can appear years later as a result of multiple factors interacting over time [10, 63]. Further to this, very few studies focused on measuring the relationship between network effects and smoking according to SES.

## Conclusions

Overall, effects according to SES were underreported in the included studies. In studies that did measure SES, variance in network characteristics and position and their association with smoking varied both between schools of a differing and those of a similar socioeconomic composition. No consistent evidence of change after the introduction of a comprehensive smoking ban was observed. Conclusions can be drawn from this review whilst ensuring that contextual factors, such as disparate methods, focus and population, are taken into account. The main conclusion is the importance of analysing differences according to SES at the organisational- and individual-level as well taking into account other contextual differences, such as school-level smoking prevalence. Results indicate that interventions would benefit from being designed to allow for adaptation according to context, with further research required to investigate what type of adaptation may increase effectiveness interventions within both differing and similar socioeconomic contexts [62].

Further network analyses are required utilising more recent data and clearly reporting differential effects. This would help to obtain a comprehensive understanding of how network processes may influence smoking differently according to SES after the introduction of a comprehensive smoking ban, and how adaptation could be used to enhance intervention effectiveness. In addition, the forthcoming sister review of qualitative findings focused on SES and peers and their relationship to adolescent smoking will help to obtain a greater insight into the context surrounding the role of peers and SES in adolescent smoking [23].

## Supplementary Information

**Additional file 1.** Glossary of social network terms. This additional file defines the social network terms used throughout this manuscript.

**Additional file 2.** Medline search strategy. This additional file outlines the search strategy for Medline.

**Additional file 3.** Risk of bias (quality) assessment. This additional file includes a table of the risk of bias assessment for each study included in the review.

**Additional file 4.** Data extraction sheet. This additional file includes a table of the key characteristics for and the key information included within each study included in the review.

## Data Availability

Further data and materials, including the quality assessment table and the data extraction database, are included in the additional files.

## Abbreviations

ASSIST: A Stop Smoking In Schools Trial
ASSIA: Applied Social Sciences Index and Abstracts
CINAHL: Cumulative Index to Nursing and Allied Health Literature
ERIC: Education Resources Information Center
ERGM: Exponential Random Graph Models
ESFA: European Smoking Prevention Framework Analysis
FINEdu: Finnish Educational Transitions
NA: Not Applicable
PECO: Population Exposure Comparator Outcome
PRISMA: Preferred Reporting Items for Systematic reviews and Meta-Analyses
PROSPER: Promoting School-Community-University Partnerships to Enhance Resilience
PROSPERO: Prospective Register of Systematic Reviews
SABM: Stochastic Actor-Based Models
SES: Socioeconomic Status
SILNE: Smoking Inequalities – Learning from Natural Experiments
SNA: Social Network Analysis
SAOM: Stochastic Actor-Oriented Models
SURE: Specialist Unit for Review Evidence
ThiS: Teenage Health in Schools study
UK: United Kingdom
