# Supplementary material for: Exploring the association between school-based peer networks and smoking according to socioeconomic status and tobacco control context: a systematic review
Source: BMC Public Health. 2022 Jan 20;22:142. doi: 10.1186/s12889-021-12333-z (PMC8772141; doi:10.1186/s12889-021-12333-z)
Supplement: Supplementary file 1 — Additional file 1. Glossary of social network terms. This additional file defines the social network terms used throughout this manuscript. [file 12889_2021_12333_MOESM1_ESM.docx]

**Glossary of social network terms**

**Social networks:** connections between individuals or groups and the social structure that this creates can be measured empirically.

**Social influence:** the level to which an individual’s smoking behaviour is directly or indirectly influenced by their peers’ behaviour and/or attitudes

**Social selection**: refers to an individual choosing friends according to whether they smoke or not. In this case smoking may initially drive friendship formation, before being reinforced through these friendships.

**Network position:** describes an individual’s position within a network, such as their level of popularity (centrality), isolation or group (clique) membership.

**Peer group structure:** the regularised patterns of interactions among adolescents in a social system.

**Homophily:** the extent to which individuals are similar to each other.

**Clique:** an exclusive group of people who share interests, views, purposes, or patterns of behaviour.

**Liaison:** a person who bridges communications between two or more groups.

**Isolates:** those who do not actively participate in cliques or friendship groups.

**Density:** the total number, compared to the total possible number of relationships in a network.
