# Supplementary material for: Exploring the association between school-based peer networks and smoking according to socioeconomic status and tobacco control context: a systematic review
Source: BMC Public Health. 2022 Jan 20;22:142. doi: 10.1186/s12889-021-12333-z (PMC8772141; doi:10.1186/s12889-021-12333-z)
Supplement: Supplementary file 2 — Additional file 2. Medline search strategy. This additional file outlines the search strategy for Medline. [file 12889_2021_12333_MOESM2_ESM.pdf]

<u>**A mixed method systematic review of associations between school-based peer networks and smoking according to socioeconomic status and tobacco control context**</u>

Quantitative Medline strategy

1 smoking/
2 exp Cigarette Smoking/
3 smoking.ti,ab.
4 ((smok* or tobacco or cigarette* or nicotine or substance) adj3 (addict* or use* or usage or using or intake or consum*)).ti,ab.
5 ((smok* or tobacco or cigarette* or nicotine) adj3 (prevalence or behavio?r)).ti,ab.
6 or/1-5
7 exp schools/
8 secondary schools/
9 ((middle or junior high or high or secondary or secondary) adj3 (school or educat*)).tw.
10 or/7-9
11 exp Adolescent/ or exp Minors/ or exp Young Adult/
12 (student* or pupil* or young adult* or young person or juvenile* or young people).tw.
13 (adolesc* or teen* or minor* or boy* or girl* or youth*).tw.
14 or/11-13
15 social networking/
16 Peers/ or peer relations/ or peer influence/ or peer group/ or friends/ or friendship/ or interpersonal relations/
17 social behaviour/ or social capital/ or social isolation/ or social norms/ or social groups/
18 social behavio?r.tw
19 ("social network*" adj2 (analys* or method* or approach or process* or influence*)).ti,ab.
20 (central* adj (closeness or betweenness or degree)).ti,ab.
21 (social network* or network or density or isolate* or clique* or liaison* or gatekeeper*).mp.
22 (school-based network or network structure).tw.
23 ((peer* or friend*) adj3 (pressure or influence or selection or norm* or role or network*)).tw.
24 or/15-23
25 6 and 10 and 14 and 24
26 (network* adj5 smoking).tw.
27 25 or 26
28 limit 27 to yr="1997 -Current"
