# Supplementary material for: Exploring the association between school-based peer networks and smoking according to socioeconomic status and tobacco control context: a systematic review
Source: BMC Public Health. 2022 Jan 20;22:142. doi: 10.1186/s12889-021-12333-z (PMC8772141; doi:10.1186/s12889-021-12333-z)
Supplement: Supplementary file 3 — Additional file 3. Risk of bias (quality) assessment. This additional file includes a table of the risk of bias assessment for each study included in the review. [file 12889_2021_12333_MOESM3_ESM.docx]

Table of risk of bias (quality) assessment

| **Author name and year** | **Study design clearly stated?** | **Clearly focused question?** | **Setting, location and relevant dates provided?** | **Participants fairly selected?** | **Participant characteristics provided?** | **Measures of exposures and outcomes appropriate?** | **Was bias considered?** | **Description of how study size was arrived at?** | **Statistical methods well described?** | **Longitudinal: Information on participant flow? OR Cross-sectional: Information on participant eligibility?** | **Results well described?** | **Sponsorship/ conflict of interest reported?** | **Limitations identified?** | **Rating (L = LOW, M = MEDIUM, H = HIGH)** |
| --- | --- | --- | --- | --- | --- | --- | --- | --- | --- | --- | --- | --- | --- | --- |
| Mercken et al. (2007) | Yes | No - question omitted but hypotheses of what expect to find. | Partly- just states about random selection of communities and then schools preference to control and intervention. Only control used as intervention was effective. | No - not randomly selected schools. | Yes - sample characteristics given in narrative and attrition analyses were performed separately for each of the three waves. | Yes - Behaviour and network questions given (no use of validated scales). | Yes - schools assigned to the experimental or control condition according to their own preference. | Partly - just states all students present on day completed questionnaires at each wave. | Yes - Model development and statistical analyses are described. Possible school effects were analysed by including eight dummy variables into the model as covariates. Since no significant school effect was found, dummy variables were excluded for further analyses. | Partly - Narrative and attrition analyses for participants. | Yes - Clear narrative with coefficients and p values. | Partly - Funding acknowledged but no information on conflict of interests. | Yes - School assignment not random but by preference, only nominate 5 same grade and school friends, and self report smoking, and limits to using SEM. | **M** |
| Mercken et al. (2009a) | Yes | No- question omitted but hypotheses defined. | Partly- just states about random selection of communities and then school allocation preference. Only control group included. | No- schools not randomly selected | Yes- sample characteristics provided and attrition analyses performed separately for each wave. | Yes- behaviour and network questions given (not validated scales) | Yes- schools allocated to control or intervention according to preference. | Partly- states that all students present on day completed questionnaires at each wave. | Yes- model development and statistical analyses undertaken are described. Possible school effects were analysed by including eight dummy variables. Since no school effect was found, dummy variables were excluded for further analyses. | Partly- narrative and attrition analyses for participants. | Yes- clear narrative with coefficients and p values. | Partly- funding acknowledged but no information on conflicts of interest. | Yes- school allocation not random, name generator limited to 5 friends and limits to using SEM. | **M** |
| Mercken et al. (2009b) | Yes | No- aims but no clearly defined questions. | Yes- setting, locations and time to follow-up are clearly stated. Signposted to the protocol paper for date of data collection | Yes- all students who were present on the day of data collection invited to participate. | Yes- smoking behaviour, age, gender, ethnicity displayed in a table. | Yes- behaviour and network measure clearly stated but not validated measures. | Yes- data were corrected if participants entered study at a later date, schools with an autocorrelation lower than 0.1 were excluded. | Yes- details on response rate broken down by country. | Yes- detailed description of analysis provided. | No- no detail on follow-up attrition. | Yes- narratives are clear and tables show values. | Partly- funding acknowledged but conflict of interest not addressed. | Yes- self-report, fixed name generator, bounded by grade, differences by classroom, school and country not explored | **M** |
| Mercken et al. (2010a) | Yes | Yes- 4 clear questions; selection, influence, reciprocation and change over time. | Yes- schools were control schools for interventions and dates were clear. | Partly- Regions randomly selected but unclear for schools. All students in seventh grade at baseline included. | Yes- table of baseline descriptive statistics provided. | Yes- network and behaviour measures given. No validated measures for behaviour. | Yes- included a number of alternative explanatory mechanisms to counter biased estimations of selection and influence processes. | Yes- all control schools that were available and all those in seventh grade at baseline. | Yes- Clear description of SABM analysis. Includes characteristics of the current network and individual attributes as control variables. | No- Unclear how many participants at each wave. | Yes- Clear results and values provided in narrative and tables. | Partly- funding acknowledged but conflict of interest not addressed. | Yes- self report smoking measures, Name generator limited to 5 friends, standardised effect sizes for SABM are not defined already, cannot rule out other extraneous variables | **H** |
| Mercken et al. (2010b) | Yes | No- question omitted but hypotheses defined. | Yes- only included control schools that took part at all 4 time points and had a minimum of 20 males and females. | Partly- Regions randomly selected but unclear for schools. All students in seventh grade at baseline included. | Yes- table of baseline descriptive statistics provided. | Yes- network and behaviour measures provided. No validated measures for behaviour. | Yes- controlled for several alternative processes explaining peer selection. | Yes- all control schools that were available and all those in seventh grade at baseline. | Yes- Clear description of SABM analysis. Includes characteristics of the current network and individual attributes as control variables. | No- Unclear how many participants at each wave. | Yes- Clear results and values provided in narrative and tables. | Partly- funding acknowledged but conflict of interest not addressed. | Yes- self report smoking measures, only Helsinki, bounded at school grade, no control for parental influence, some constructs only used 1 question, no comparison across waves. | **H** |
| Turner et al. (2006) | Yes | Yes- 4 questions linked school differences in smoking rates. | Yes- purposively sampled pair of schools with high and low smoking rates but similar SES composition. Date and school characteristics provided. | Yes- all students in these schools aged 13 and 15. | Partly- data about gender and age provided in table. | Yes- behaviour and network measure clearly stated but not validated measures. | No | Yes- number of those who did not consent provided. | Partly- brief overview of what was tested. | Cross-sectional: Yes- response rate provided. | Yes- narrative clear and tables display values. | Partly- funding acknowledged but conflict of interest not addressed. | Yes- 2 schools and only low SES, cross-sectional, limited to reciprocal relationships. | **L** |
| Pearson et al. (2009) | Yes | No- aim, but no clearly defined question. | Partly- some details provided. Only schools with identified school effects on substance use and selected to identify 2 schools with differing smoking prevalence but similar SES. | Partly- all students aged 13 and 15 in selected schools. | Partly- descriptive statistics provided as functions of sociometric measures, rather than whole sample. | Yes- behaviour and network measure clearly stated including how UK standard measures used for smoking and SES. | No | Yes | Partly- very brief summary of statistical analysis. | Cross-sectional: Partly- all students aged 13 and 15 in selected schools. Data on non-consent and missing data provided. | Yes- clear results given in narrative and tables with values. | Partly- funding acknowledged but conflict of interest not addressed. | No- findings are from a single, cross-sectional study but do not discuss limitations. | L |
| Steglich et al. (2009) | Yes | No- aims, but no clearly defined question. Exploratory. | Yes- purposive sampling of schools is detailed to give a range. Dates, locations and school characteristics given. | Yes- Cluster randomised trial with the whole of Year 8 involved. | Yes- sample characteristics provided for each school. | Yes- behaviour and network measure stated but not validated measures. | No. | Yes- purposive sampling of schools and a larger sample not considered valuable due to the technical nature of questions. | Yes- very detailed description of statistical analyses. | No | Yes- very detailed results given. Summarised in narrative, but values given in tables. | Partly- funding acknowledged but conflict of interest not addressed. | Partly- less about limitations but what analyses will be performed in the future. | **M** |
| Mercken et al. (2012) | Yes | Yes- 3 clear questions; influence, selection and change over time. | Yes- school selection from control schools available including country given. All Year 8 students involved. | Yes- cluster randomisation used in intervention. | Yes- table of descriptive statistics provided. | Yes- network, behaviour and SES measures provided. SES used Family Affluence Scale as a validated measure. | Yes- cotinine used to validate self-reported smoking. Data used to assess amount of misreporting, not to correct data. Attrition analysis verified no significant difference for drop-outs. | Yes- school selection from control schools available including country given. All Year 8 students included. | Yes- Clear description of SABM analysis. Includes characteristics of the current network and individual attributes as control variables. | Yes- table of statistics and attrition analyses provided. | Yes- clear results for each research question given in narrative and tables. | Partly- funding acknowledged but conflict of interest not addressed. | Yes- SES scale used, network bounded by school, smoking behaviour assessed with one question and need to examine quitters in future. | **H** |
| Copeland et al. (2017) | Yes | No- question omitted but gives 4 hypotheses with clear link to literature. | Yes- school districts given, how schools were sampled and grades for each cohort. | Yes- gives details of those omitted from analyses and why. | Yes- table of descriptive statistics provided. | Yes- details measures and how students were assigned to isolation categories. | No | Yes | Yes- controlled for and dummy variables given and removed those without longitudinal data. | No | Yes- clearly outlines results for each hypothesis in written text and effect sizes and significance level are provided in table. | Partly- funding acknowledged but conflict of interest not addressed. | Yes- rural sample, those anti-social less likely to thoroughly complete survey, other measures of delinquency could be used and limited data on external orientation. | **M** |
| Ragan (2016) | Yes | No- clear aims and objective, but no question. | Partly- dates given but not sure how districts and schools were selected. | Partly- all students in 6th grade involved. | No- not clear from table of sample characteristics. | Yes- behaviour and network measure clearly stated but not validated measures. | No | No | Yes- modelling strategy clearly described and covariates to control for other factors that may influence cigarette use and beliefs about smoking outlined. | No- states an average of 6,200 students at each wave and more than 9000 students overall are analysed. | Yes- clear results given in narrative and tables with values. | Partly- funding acknowledged but conflict of interest not addressed. | Yes- sample generalisability, network bounded by school and grade and model issues outlined. | **M** |
| McMillan et al. (2018) | Yes | No- key aim, but no question. | Partly- gives details at the district level but not school. Dates given from larger study, PROSPER. | No- data not clear on participant recruitment. | Yes- gives grade, gender and delinquency by wave and overall. | Yes- smoking and network measures are standard and delinquency measure draws on previous studies. | Yes- In SAOM structural effects controlled for in friend selection and individual behaviour estimates. Some networks were excluded as they did not reach convergence | Partly- no detail on size of eligible sample, but gives high response and retention rates. | Yes- addresses why used and what was included in SAOM. Gives reasons for omitting schools. Control network measures described. Sensitivity check showed results still held when limited to networks that achieved convergence. | Yes- narrative described responses and retention rates overall but not individually for each wave. | Yes- narrative and tables easy to follow showing clear b and p values. | Partly- funding acknowledged but conflict of interest not addressed. | Yes- name generation limited to 7 friends and bounded by grade. Large, urban communities under represented and issues with implying causality. | **H** |
| Osgood et al. (2014) | Yes | No- aim, but no clearly defined question. | Partly- information about the larger study, PROSPER, provided. Describes how districts selected but no school data. | Partly- Describes how districts selected but no school data. All students in 6^th^ grade eligible. | Yes- narrative and table of descriptive statistics including gender, ethnicity and SES in sample as a whole and in each category. | Yes- positions, smoking and control variables defined. Not validated measures. | No | No | Yes- clear description of analysis, refining traditional tripartite division of group positions and controlling for factors associated with adolescent substance use and representing alternative explanations. | No- just states that there were approximately 9,500 per wave. | Yes- clear results provided in narrative and tables with values. | Yes- no conflicts declared and funding source described. | Yes- population used and causation. | **M** |
| Ennet et al. (2008) | Yes | No- question not defined but overall aim given. | Partly- dates given and all schools in 3 school systems eligible. | Yes- provides details of those eligible and reasons why some participants were omitted. | Yes- sample descriptive statistics provided in narrative. | Yes- Smoking involvement used validated smoking scale. Outlined friendship and network measures and informed by literature. | No | Yes | Yes- thorough description given in analysis section. Multiple imputation used for missing values as participants entered and left at different waves. Control variables described. | Yes- no clear flow of individuals but percentages which completed the number of waves given. | Yes- clearly outlines finding from each model using B vale and p-values. | Partly- funding acknowledged but conflict of interest not addressed. | Yes- cannot assess temporality of relationships and infer selection or influence. Did not assess whether friend smoking predicted later smoking. | **M** |
| Ennet et al. (2006) | Yes | No- overall purpose and hypotheses given. Hypotheses do not specify nature of relationship between variables. | Partly- dates given and all schools in 3 school systems eligible. | Yes- gives details of those eligible and reasons why some participants were omitted. | Yes- sample descriptive statistics provided in narrative. | Yes- information on substance use, demographics and network measures at adolescent and network level given. Some reference to literature on adolescent network measures. | No | Yes | Yes- thorough description provided in analysis section. | Yes- no clear flow of individuals but percentages which completed the number of waves given. Imputation of missing data for reciprocity in friendship dyads. | Yes- description of adolescent level and network level stats given before relationships between network level and substance use. Narrative gives results with ORs and p levels. | Partly- funding acknowledged but conflict of interest not addressed. | Partly- gives limitations to analyses but not other areas, such as sample size. | **M** |
| DeLay et al. (2013) | Yes | No- aim, but no clearly defined question. | Yes- country and dates provided and school setting described. | Yes- gives details of school exclusion due to low participation and tests for assessing missing data. | Yes- a written narrative of the sample is given, but no table. Descriptive statistics for the network and smoking are given. | Yes- two measures of sociometric and behaviour data included. Control effects used in model. | No | Yes- including how schools were omitted due to low participation rates. | Yes- details how participants missing sociometric and behavioural data were included but calculations minimise their contribution to parameter estimates. | Yes- Provides numbers for each wave and examined students from participating schools did not differ from excluded schools. Checks were made to ensure missing data were random. | Yes- clearly outlines selection, deselection and socialisation effects with associations in narrative and full details in table. | Partly- funding acknowledged but conflict of interest not addressed. | Yes- only 2 timepoints, age of students in sample, smoking measures and issues with analysis with not looking at multiple network membership. | **H** |
| Kiuru et al. (2010) | Yes | No- aim, but no clearly defined question. | Yes- dates and school details provided. | Yes- gives details of grades eligible and why some schools were omitted. | Partly- only age provided. | Yes- behaviour and network measures given but not validated. | Partly- highlights self-report measures but not how this bias was mitigated. | Yes- Only schools with over 65% participation at both waves. | Yes- very comprehensive section on statistical methods used, including SABM, multi-level models and comparison of peer group to whole network. Missing at random approach was applied and 4 schools were removed due to low participation rates. | No- gives participation rates in school but no participant flow. | Yes- written narrative clear about what was shown and about where in tables statistics were given. | Partly- funding acknowledged but conflict of interest not addressed. | Yes- participants older adolescents, only used same grade peers, name generator limited to 3 and self-report. | **H** |
| Huisman & Bruggeman (2012) | Yes | Yes- core research question clearly stated. | Partly- dates provided but not clear how schools were selected. | Yes- selected grade/age of participants based on national level data on when smoking onset occurs. | Yes- descriptive statistics provided in table. | Yes- network measure outline, smoking assessed but not through validated scale. Other variables include parental educational level and school type. | No | Yes | Yes- missing network data checked to be below SABM threshold from other studies. | Partly- absenteeism given for both waves, but not individual flow of participants. | Yes- RSiena coefficients and p value provided in table and narrative provided explains overall results. | No | Yes- limitations given- issues with RSiena, self-report data, and network bounded by grade, rural schools only. High level of unknown and missing data on parental education level. | **M** |
| Huisman (2014) | Yes | Yes- core research question clearly stated. | Partly- dates given, but not clear how schools were selected. One school omitted due to high level of missing friendship and behaviour data. School characteristics given. | Yes- selected grade/age of participants based on national level data on when smoking onset occurs. | Partly- school characteristics given. | Yes- network measure outline, smoking behaviour and attitudes assessed but not through validated scale, other variables include school type, gender and age. | No | Yes | Yes- methods described adequately including using 2 models; one each for attitude and behaviour. One school omitted due to high level of missing data on friendship and behaviour. Other 4 checked for missing data to be below SABM threshold. | Partly- absenteeism given for both waves and combined, but no individual flow of participants. | Yes- RSiena coefficients and p value given in table and narrative provided explains overall results. | No | Yes- network bounded by school, only rural areas, self-report smoking data, and limitations to using RSiena. | **M** |
| Lorant et al. (2017) | Yes | Yes- 2 clear questions | Yes- dates, cities and stratification of schools given. | Yes- gives number of and reasons for both school and individual non-participation. | Yes- table of sample gender, SES, city and smoking status provided. | Yes- used 2 smoking measures for dependence and frequency. Also used a variety of SES measures. | No | Yes- shows breakdown of who participated and reasons for non-participation. | No- not much depth to data analysis and only regression used. Removed participants without complete data and verified that those that did not participate did not differ from those who did. | Yes | Yes- the narrative is easy to follow and tables display odds ratios and p values. | Partly- funding acknowledged but conflict of interest not addressed. | Partly- cross-sectional data, checks on internal and externalvalidity, but nothing about measures. | **M** |
| Robert et al. (2019) | Yes | Yes- 3 clear questions | Partly- date, cities and school types given. No data on why schools did not participate and differences to those that did participate. | Partly- not clear if all students or just certain grades were eligible. Does provide number approached and consented. | Yes- table of descriptive statistics provided. | Yes- behaviour and network measure stated but not validated measures. | No | Partly- not clear if all students or just certain grades eligible, but gives number approached and consented. Observations with missing data were removed. | Yes- data analysis overview given. Covariates included the number of smokers among family members in the household, SES, age and sex. Excluded observations due to missing data. | Yes | Yes- clear results given in narrative and tables with values provided. | Yes- It was reported that there were no conflicts and funding source was declared. | Partly- self-report for academic performance, differences across countries for educational systems, cross-sectional design, but nothing about measures. | **M** |
| Mulassi et al. (2012) (cross-sectional) | Yes | No- question omitted but objective of study given. | Partly- location and dates provided, but not clear why the 1 school was selected. | Partly- all students in 1 school in 2^nd^ to 5^th^ year, but not clear why this school was selected. | Yes- short description of sample in narrative. | Yes- network and behaviour measures provided. Validated scales not used for behaviour. | No | Partly- all students in 1 school in 2^nd^ to 5^th^ grade. | Partly- short summary on tests used but unclear whether control variables used. | Partly- All students in 1 school in 2^nd^ to 5^th^ year. | Yes- clear results given in narrative with values and figures. | Partly- no conflicts were declared. | Partly- sample size, age range and obesity measure were considered, but not other issues such as self-report and statistical analysis used. | **L** |
| Valente et al. (2013) (but ERGM) | Yes | Partly- two clear questions and an aim. | Yes- date and district data given but not sure about school characteristics. | Yes- clear description of eligibility numbers, parental consent, student assent and final numbers. All students in 10^th^ grade eligible. | Yes- risk behaviour rates, socio-demographic characteristics and in-degree scores for the sample. | Yes- behaviour and network measure clearly stated but no validated measures. | No | Yes- clear description of eligibility numbers, parental consent, student assent and final numbers. | Yes- process described and covariates normally associated with smoking and drinking were included; age, sex, ethnicity, free school lunch, number of rooms in household, academic achievement, parental and sibling smoking/drinking. | Yes- clear description of eligibility numbers, parental consent, student assent and final numbers. | Yes- narrative are clear and tables show values. | Partly- funding acknowledged but conflict of interest not addressed. | No | **M** |
| Forster et al (2015) | Yes | No- aim and hypothesis, but no clearly defined question. | Partly- data collection date not specified, but provides community characteristics. | No- classroom selected by teachers within the school and some did not obtain parental consent. May not be representative of school. | Yes- some sample characteristics given by whole sample and separately for isolates. | Yes- outlines measures used including demographics, social network measures, substance use, parental supervision. Validated scales include social self-control, interpersonal aggression, violence exposure. | Yes- considers recall and attribution bias as a limitation of using self-report measures but doesn’t account for it. | Yes | Yes- described well but also uses diagnostic analyses to look at overdispersion. | Yes- selected classes by teacher and some parents did not consent. | Yes- results are succinct but p values provided in narrative. | Partly- funding acknowledged but conflict of interest not addressed. | Yes- Limited sample size, cross-sectional design, self-report measures and non-representative school sample. | **L** |
| Hall & Valente (2007) | Yes | No- none stated. | No- school-based but information on location or date of data collection was missing. | No- schools were selected according to availability of smoking and network data from a previous round of data collection. Number of classrooms and participants reported, but no information on % that provided parental consent. | Partly- no table, but mean age, ethnicity and gender were described in text. | Yes- behaviour and network measure clearly stated, but no validated measures. | Partly- missing data were imputed. | No- no detail on response rate. | Yes- detailed description provided and classroom size was accounted for. | No | Yes- narrative is clear and tables provide values. | No | Partly- just that the network were bounded by classroom. | **M** |
| Ramirez-Ortiz et al. (2012) | Yes | No- aim provided but no clearly defined question. | Partly- dates and information about location. High risk cohort specified. | Partly- invited first and second semester students to participate. | Partly- only information on age and whether working and studying provided. | Yes- behaviour and network measure stated but not validated measures. | Yes- follow-up attrition was reported. | Yes- eligibility and attrition given. | Partly- a brief overview of analyses provided. | Yes. | Yes- brief but clear results provided in narrative and tables with values. | Partly- funding acknowledged but conflict of interest not addressed. | Yes- attrition, smoking measure and only within school, but nothing about analyses used or sample size. | **L** |
| Lakon & Valente (2012) | Yes | No- question omitted but hypotheses of what will be found as a high risk sample. | Partly- recruitment and dates given and reasons districts were omitted. Does not give details of schools, but districts. | Partly- certain classrooms were selected, but criteria unclear. Purposive sampling undertaken of continuation high schools to address high risk population. | Yes- overview of sample age, ethnicity and maternal education, as well as numbers in each smoking category were provided in narrative. | Yes- behaviour and network measure details- no validation of smoking measure. Detailed account of how each network measure was constructed. | Yes- 65.5% participation rate (513 students without valid consent so not involved). | Yes- gives breakdown of those eligible, those omitted and why. Reasons included parental consent, missing smoking data and class size. | Yes- explains methods and why chosen (i.e. nesting of students in classrooms). Used multiple imputation and explains why. | Yes | Yes- narrative gives clear overview of main results and effect, as well as p values. | No | Yes- high risk population used so not generalisable to mainstream, cross-sectional data, name generation limited to 5 friends. | **M** |
| Van Ryzin et al. (2016) | Yes | Yes- 2 clear questions. | Yes- dates of surveys, district and some school characteristics. | Yes- provides response rate and % that completed all waves. Data on individual waves not provided. A students in sixth grade at baseline. | Yes- descriptive statistics provided and broken down by school. | Yes- behaviour and network measure clearly stated but not validated measures. | No | Partly- provides response rate and % that completed all waves. Data on individual waves not provided. | Yes- detailed description of statistical analyses provided. | Partly- provides response rate and % that completed all waves. Data on individual waves not provided. | Yes- narrative are clear and tables display values. | Yes- It was reported that there were no conflicts and funding source was declared. | Partly- limitations to SABM and RSiena and to sample characteristics. No details about self-report measures or missing data. | **M** |
| Valente et al. (2005) | Yes | Yes- 3 clear questions. | Yes- dates of surveys, district, and school characteristics provided. Verified that no significant differences between those refusing and participating schools. | Yes- clear description of eligibility numbers, parental consent, student assent and final numbers. All students in 6^th^ and 7^th^ grade eligible. | Yes- table of descriptive statistics provided. | Yes- behaviour and network measure clearly stated but not validated measures. | No | Yes- clear description of eligibility numbers, parental consent, student assent and final numbers. | Yes- limited data on logistic regression and variables controlled for. Missing data checked through comparison between different samples. | Yes | Yes- narrative are clear and tables show values. | Partly- funding acknowledged but conflict of interest not addressed. | Partly- noted sample ethnicity and location so cautious about generalisability. No details on self-report or limited name generation. | **M** |
| Kobus & Henry (2010) | Yes | Yes- 2 clear questions. | Partly- setting and location provided. Date of data collection omitted. | Yes- all students in 6^th^, 7^th^ and 8^th^ grade eligible. | Yes- Descriptive statistics provided and displayed in a table. | Yes- behaviour and network measure clearly stated but not validated measures. | Partly- 2 participants were excluded due to reporting that they lied in their survey responses. Study did not consider friends outside of school. | Yes- all students in 6^th^, 7^th^ and 8^th^ grade eligible. Provides % of students in attendance and % who opted out. | Yes- detailed description of analyses provided. | Yes- eligibility and response rate provided. | Yes- narrative are clear and tables display values. | Partly- funding acknowledged but conflict of interest not addressed. | Partly- study design, definitions of network positions, heterogeneity within network positions not explored. No details on self-report, missing data or analysis method. | **M** |
